# Dr. Heermann

**Published:** 1833

**Authors:** 


Lewis Heermann, M. D. Doctor Heermann was a native of Germany, but had been in the Medical Department of our navy, for more than 25 years. For a long time he was hospital surgeon at New Orleans. Two or three years since he was appointed Fleet surgeon in the Mediterranean service, but a severe illness compelled him to return to the United States; when by regular promotion he became the senior surgeon of the Navy, a rank which he held at the time of his death, which was caused by the Cholera in New Orleans.

The deceased was a man of respectable talents, and extensive professional acquirements; an excellent disciplinarian, and an accomplished gentleman. We knew him personally, and esteemed him for his precision of manners, and animated conversation; but are without the materials for such a tribute to his memory, as his virtues and official rank unite in demanding for him.

At present, we can only add, that when a young man, he was present with Commodore Decatur, and assisted in the burning of the frigate Philadelphia, in the Mediterranean.

